# Experiences of Frontline Managers during the COVID-19 Pandemic: Recommendations for Organizational Resilience

**DOI:** 10.3390/healthcare12030407

**Published:** 2024-02-04

**Authors:** Sonia Udod, Pamela Baxter, Suzanne Gagnon, Gayle Halas, Saba Raja

**Affiliations:** 1 Helen Glass Centre for Nursing, College of Nursing, Rady Faculty of Health Sciences, University of Manitoba, 89 Curry Place, Winnipeg, MB R3T 2N2, Canada; saba.raja@umanitoba.ca; 2 School of Nursing, Faculty of Health Sciences, McMaster University, 1280 Main St. W, Hamilton, ON L8S 4L8, Canada; baxterp@mcmaster.ca; 3 I.H. Asper School of Business, University of Manitoba, Winnipeg, MB R3T 5V4, Canada; suzanne.gagnon@umanitoba.ca; 4 Rady Faculty of Health Sciences, University of Manitoba, P228—770 Bannatyne Ave., Winnipeg, MB R3E 0W3, Canada; gayle.halas@umanitoba.ca

**Keywords:** nurse manager, COVID-19, crisis, leadership, nurses, nursing workforce, working conditions, workplace resilience

## Abstract

The COVID-19 pandemic caused a global health crisis directly impacting the healthcare system. Healthcare leaders influence and shape the ability of an organization to cope with and recover from a crisis such as the COVID-19 pandemic. Their actions serve to guide and support nurses’ actions through unpredictable health service demands. The purpose of this paper was to examine frontline managers’ experiences and organizational leadership responses that activated organizational resilience during the COVID-19 pandemic, and to learn for ongoing and future responses to healthcare crises. Fourteen managers participated in semi-structured interviews. We found that: (1) leadership challenges (physical resources and emotional burden), (2) the influence of senior leader decision-making on managers (constant change, shortage of human resources, adapting care delivery, and cooperation and collaboration), and (3) lessons learned (managerial caring behaviours and role modelling, adaptive leadership, education and training, culture of care for self, and others) were evidence of managers’ responses to the crisis. Overall, the study provides evidence of managers experiences during the early waves of the pandemic in supporting nurses and fostering organizational resilience. Knowing manager’s experiences can facilitate planning, preparing, and strengthening their leadership strategies to improve work conditions is a high priority to manage and sustain nurses’ mental health and wellbeing.

## 1. Introduction

The World Health Organization declared the novel coronavirus, COVID-19, to be a global pandemic on 11 March 2020 [1]. The urgent need to respond to the pandemic placed unprecedented demands on healthcare systems in Canada and globally by challenging regional and organizational capacity for containing the outbreak, rationing of healthcare supplies, infection control, and information management, placing intense pressure on frontline managers and staff to make difficult decisions while working under demanding physical and psychological stress. The sequelae of these pressures are still evident among the healthcare workforce and the healthcare environment.

Few empirical studies existed on healthcare leadership in crises prior to the pandemic. However, the literature was often supplemented with anecdotal lessons learned and corrective action planning [2,3]. Currently, the role of frontline managers during the crisis is under investigation. The role of a manager is to focus on the emotional wellbeing of their staff and the critical importance of effective communication [4], and the need for courageous leaders with sound knowledge [5,6]. Others have argued for the need to rely on distributed leadership [7] with empowerment being central during crisis [8]. What is notable is that rising workloads, insufficient staffing, as well as exhaustion and burnout, were serious concerns for nurses and nurse leaders before the crisis, and made worse with the prolonged pandemic [9,10]. Managers face many challenges as a result of the pandemic including physical and psychological health problems [11]. Equipping healthcare leaders with the capabilities and resources to manage all aspects of their responsibility, including one’s own emotional health is a requisite priority for the ability to lead in a crisis.

### 1.1. Crisis Leadership

The pandemic has further challenged leaders’ abilities to solve problems and make rapid decisions in reacting and adapting to the crisis [12,13,14,15]. Crisis leadership implies an integrative approach that fosters collective action and caring relationships while promoting clear communication and a clear vision to achieve a common good [16,17]. Effective crisis leaders create psychological safety by being empathetic to how people react to loss and uncertainty and enable individuals to cope and recover from a health-related crisis [16,17,18,19]. 

The effectiveness of leadership styles depends in part on the nature and stage of the crisis, what led to the crisis, and how leaders interacted with others in the organization in preparing for the possibility of an organizational crisis [3,20]. Waldman et al. (2001) found that during high uncertainty, charismatic leadership behaviours which communicate determination and articulate high-performance expectations are predictive of positive organizational performance [21]. These findings are supported by Halverson (2004) who found self-sacrificing behaviour in leaders was judged positively by team members during a crisis but was perceived negatively when uncertainty was absent [22].

### 1.2. Fostering Organizational Resilience

Leadership is critical in a crisis and affects organizational resilience [16,17]. The study of resilience may provide a more complete understanding of the relationship between a crisis and the organization [17]. At the organizational level, resilience refers to an organization’s ability (through resources, ideologies, structure, routine) to react to and recover from a disturbance with minimal effects on its stability and function [23,24]. Organizational resilience includes the interaction between an organization, its stakeholders, and the environment while confronted with a crisis [17]. In other words, there are certain features of a system (work relations and culture) that play a role in how staff within that system experience and respond to a crisis.

Havaei et al. (2021) found less favourable workplace conditions for nurses such as organizational support and relationships, organizational preparedness, workplace safety, and access to supplies and resources linked to adverse mental health outcomes [2]. Leader behaviour can directly impact staff, quality of patient care, and organizational performance [20,25]. Considering ongoing transformations in healthcare environments to improve the delivery of care, and the compounding stress of the COVID-19 crisis, organizational resilience is a high priority facing frontline managers in Manitoba, Canada.There is a need to understand how leaders foster organizational resilience, and identify key lessons that can be applied in many other jurisdictions.

To address the gap, the aim of the overarching study sought to understand from frontline managers how they activated organizational resilience in the healthcare setting during the COVID-19 crisis response and to learn for ongoing and future responses to healthcare crises. In this paper, we address frontline managers’ experiences and organizational leadership responses during the COVID-19 pandemic.

## 2. Methods

### 2.1. Study Design

A qualitative design provides opportunities for deeper insight and in-depth questioning and probing to elicit managers’ experiences through semi-structured individual interviews [26] An Advisory Committee of key stakeholders including senior health leaders, directors, and frontline manager representatives from the health regions was struck at the beginning of the project to provide feedback to the research team on an ongoing basis. The goals of the committee were to support adequate recruitment of participants, provide consultation, and knowledge dissemination. 

### 2.2. Sample and Setting

Frontline managers are frontline supervisors (registered nurses or from other health-related disciplines) of clinical nurses and other care providers who have 24 h 7 days per week responsibility for their unit(s). Through criterion sampling [26], frontline managers who have experiential knowledge of leadership work in the COVID-19 crisis were recruited. Frontline managers were recruited from three health regions in Manitoba, Canada to allow for a diversity of organizational contexts and individual experiences. Ethical approval was obtained from the University of Manitoba (HS24260) and health regions, and site access approvals were obtained.

### 2.3. Participants and Data Collection

A purposeful sample of 14 frontline managers was recruited from three health regions in the province of Manitoba. There was variability of participant experiences and representation from various clinical contexts in the health regions. Members of an Advisory Committee arranged for the distribution of the recruitment emails through their organizations, described the study, and answered any further questions. Inclusion criteria for participants were: (1) being willing to share his/her experiences during the COVID-19 situation; and (2) having worked as a manager during the COVID-19 crisis. Snowball sampling was also used to identify and provide contact information for managers who met the inclusion criteria and were willing to be part of the study. An email was sent to participants by the senior leader’s administrative assistant in each health region describing the study and inviting them to discuss their experiences as frontline managers in individual semi-structured interviews. Once participants contacted the researcher and/or the research coordinator and indicated their willingness to participate, they were consented to be part of the study and also completed a questionnaire by email to provide descriptive statistics and capture socio-demographic characteristics to assess similarities and differences among various data groups.

Data collection was conducted remotely via video conferencing (Zoom or MS Teams) at a mutually agreed upon meeting time. Individual interviews were preferred as coordinating individual interviews during a pandemic was more manageable for participants and addressed safety concerns. Participants answered interview questions based on an extensive literature review and which were reviewed by the research team covering four broad areas: leadership challenges they faced, how they worked with stakeholders and how decisions were made in responding to the crisis, how interactions with managers shaped staff motivation, and lessons learned to improve organizational resilience (see Table 1). In each interview, the researcher reaffirmed participant privacy and confidentiality. Each interview lasted approximately 45–60 min depending on participant availability and was digitally audio recorded.

### 2.4. Data Management and Analysis

A thematic analysis was undertaken to explain frontline managers’ experiences and how organizational resilience was activated in the healthcare setting. Digital recordings of the individual interviews were transcribed by an experienced transcriptionist. Data was stored and analyzed using NVivo 12 qualitative software. Braun and Clarke’s six-phase approach [26,27,28] to thematic analysis was applied to enhance scientific rigor in this study and we remained open to new codes and categories emerging beyond the established domains to reflect the data. One author, SR, checked the transcripts by verifying them against the recordings. Two authors (S.U., S.R.) read and re-read transcripts to determine similarities and differences. We then debated key codes to develop initial codes, categories, and themes. We then shared the codes and themes with team members (PB, SG, GH) to review and refine the codes. As we continued coding the transcripts, we noticed that some codes were referenced by most or all of the participants. It was at that point we knew data saturation (i.e., no new concepts were being identified) had been reached. In the final phase of data analysis, the codes were refined and each theme was analytically refined to produce an overall conceptualization of managers’ experiences and organizational leadership responses during the COVID-19 crisis. Study rigor was supported by four dimensions of trustworthiness [29]. Credibility was met through member checking, with participants reviewing transcripts or providing comments to strengthen accuracy of interpretations. Dependability was met with an audit trail documenting individual interview content and theme development. Confirmability was met by independent coding and analysis of a portion of the data by the team and by an iterative feedback process until consensus was reached. Transferability will allow findings to be applied in similar contexts for healthcare leadership in disaster preparedness.

## 3. Results

As indicated in the participants’ demographics table (Table 2), all frontline managers were female and had considerable managerial experience (nearly 60% had 5 or more years of experience). Most managers were in the age ranges of 41–50 years (42.86%) and 51–60 years (35.71%) and supervised at least 51–100 people (64.29%). Most participants had a degree in nursing in addition to degrees in other disciplines (85.71%) and supervised a variety of departments and units as indicated in Table 2.

Three key themes emerged in the early waves of the pandemic from the interviews: Leadership challenges, influence of senior leader decision-making on managers, and lessons learned (see Table 3 for the narratives linked with the sub-themes within each theme).

### 3.1. Theme 1: Leadership Challenges

This theme encompassed the experiences and meaning of how frontline managers were impacted professionally and personally during the early waves of the COVID-19 pandemic. This theme was also significantly influenced by the pressures and stressors within an unprecedented context.

**Inadequate physical resources** were a major challenge faced by participants. Participants focused on gathering or advocating for personal protective equipment (PPE) while also navigating shortages of other medical supplies to keep staff and patients safe. On a broader scale, participants were involved in allocating more beds for COVID-19 patients and were involved in creating designated COVID units.

**Moral burden** from the crisis resulted in a heightened emotional burden for both frontline managers and staff. An overarching moral burden for participants was the duty to focus on safety concerns for patients, the staff’s families, and their own families. Participants witnessed the increased stress nurses faced as they provided care to patients at times with inadequate resources and under complex and fearful circumstances. Participants encountered fear and resistance from staff as they worked longer hours given additional responsibilities that emerged in the pandemic. Providing emotional support to staff who experienced patient suffering and death without family nearby was exhausting and draining. As the shock and uncertainty of the pandemic dissipated for staff, they became more anxious, exhausted, and demoralized as the intensity and pressure of providing care did not ease. Participants worked diligently to assist staff to balance family and work which at times led to staff shortages on units.

Participants faced significant pressures on a daily basis from different stakeholders—staff, patients, and health leaders to a wide range of challenges. Participants worked longer shifts and were given additional responsibilities. For example, creating more beds on their unit and responding to visitor restrictions. Participants experienced insomnia, fatigue, exhaustion, and some stayed in a remote location from their family to decrease the risk of infecting family members. This often led to feelings of loneliness that in part affected their psychological health.

### 3.2. Theme 2: Influence of Senior Leader Decision-Making on Managers

Participants experienced decision-making at various levels and in two ways: complying with directives from superiors believing that senior leaders/government were seeking the best options, and yet also making independent decisions in operationalizing directives that were not always viable or reasonable. For the most part, during the initial stages of the pandemic, senior leaders made decisions for staff at the front lines of care, seemingly without the requisite information of what was occurring on the front lines, and without being fully aware of how the consequences of their decision-making affected staff’s ability or inability to implement decisions. Change, adapting care practices, and nursing shortages heavily influenced how care was delivered.

Initially, decisions were made quickly by leaders as time was of the essence in the ever-changing situation of the pandemic. As the pandemic wore on, there was more input and exchange of information into decision-making from participants and consequently improved collaboration among managers, leaders, services, and departments.

**Constant change became a normal part of the workplace.** Constantly changing communication from superiors and from staff was a central time-consuming element of participants’ work. The flow of information was overwhelming at times, both in terms of content and frequency. Communication focused on navigating and sorting through a plethora of incoming information and guidelines about standards, infection control, and nursing care protocols. At times, information was replaced with updated guidelines or processes before reaching the intended health provider. Some information contradicted previous information, creating uncertainty and insecurity regarding what information to use. Technology became indispensable for meetings and for supporting “just in time” ways of communicating.

**Adapting the delivery of care** as a result of a rapidly changing care environment involved considerable decision-making. Guidelines established by the hospital management team were implemented to decrease the risk of staff contracting the virus, for treating infected patients, and for various other approaches to patient care but were often changed on a daily basis. Participants had some decision-making authority to adapt workflows and work procedures to the local context. For example, a lack of guidelines in some situations prompted creativity for innovative ways of delivering care without needing to work through a labyrinth of meetings and development of policies and standards. This had the added benefit of quickly solving problems on the front lines of care and highlighted the focus on the patient.

A **shortage of human resources**, especially professionally regulated nurses, to care for critically ill patients in intensive care units, emergency departments, and COVID units was a major concern. There was a concern to have nurses with the appropriate skills and knowledge, but this was not always possible. These shortcomings led to deploying nurses from other units to serve as extenders (carrying out patient care duties they felt safe and qualified to do) and training nurses from other departments in a condensed training program. Accommodations needed to be made in response to staff infected with the virus, which exacerbated staff shortages.

The crisis created an opportunity for **cooperation and collaboration** among leaders, managers, and care providers in the hospitals. There was a recognized need for mutual support within and among managers, multidisciplinary teams, and services to engage in new work processes and clarify potential misinformation in working together. Participants consistently expressed feelings of being supported that created a sense of unity with the management team, multidisciplinary teams, and staff. Cooperation and collaboration created bonds of support that facilitated agile decision-making at the front lines of care.

### 3.3. Theme 3: Lessons Learned

Despite numerous challenges and pressures participants reported strategies that enabled them to cope and respond in the COVID 19 environment. The majority of the interviews focused on managing in the moment, but several categories revealed recommendations that eased participants abilities to manage in a time of crisis.

**Managerial caring behaviours and role modelling** were demonstrated in acts of comforting and supporting staff. Participants felt a need to model a demeanour of calm, be in control of the situation and be confident in their ability to manage and lead on the unit, although not always feeling they had the capacity. It took focus and energy to not project their fear or worry onto staff, anticipate problems and/or minimize their impacts. For example, managers took time away from their administrative responsibilities in favour of taking patient assignments due to understaffing or in supporting nurses caring for dying patients without family members present.

**Adaptive leadership** was described as participants taking steps to resolve problems and make decisions without having guidelines to rely upon. Participants had flexibility and freedom to make their own decisions in response to work and staffing situation. This adaptability facilitated participants’ ability to accelerate change which was viewed as desirable to benefit patient care and support nurses.

**Training and education** on crisis management was viewed as a strategy to strengthen staff’s skillset and/or confidence in subsequent crises. Examples included having the skill and ability to carry out unfamiliar work tasks in the case of reassignment to new areas, and challenges directly related to exhaustion and burnout, and dealing with workplace violence and abuse.

Creating a **culture of care for self and staff** including physical and emotional/mental health support for themselves and staff was a key learning. Participants expressed that superiors, peers, staff, and family provided the professional and personal support required to keep going. Supportive self-care strategies including focusing on life outside work (to maintain work–life balance), exercise, leaning on personal connections, taking things one at a time, and minimizing news and social media intake. In so doing, participants relied upon their decision-making abilities, planning skills, and autonomy to care for themselves and staff.

## 4. Discussion

This study revealed that frontline managers interviewed for the study in the early pandemic had considerable leadership challenges, took opportunities to support nurses to deliver best possible care, and focused on relationships in caring for others that fostered organizational resilience.

Our findings also revealed that managers experienced stress and anxiety in assigning nurses to accomplish certain activities without the requisite resources to reasonably accomplish the activities. Additionally, managers also found that there was minimal to no input from managers or staff in the context of the need for more physical and human resources. Moral distress arises from knowing the right thing to do but organizational constraints prevent one from doing what is needed [19]. This kind of challenge was experienced by managers who had to support and convey decisions from superiors, but they knew would not be well received by nurses. This type of stress may contribute to exacerbating the turnover of nurses at the frontlines [9,30].

Managers’ initial responses to the pandemic were emotional reactions of shock and uncertainty. Mangers faced significant challenges and responsibilities that included longer work hours, responding to evolving guidelines/procedures for personal protection, staff and patient safety, creating COVID-19 beds and units, sharing constantly changing information, and supporting staff in the face of increased workloads. Our findings correlate with others, suggesting that managers and leaders focused on their own and others complex emotions in order to provide a psychologically safe workplace to care for patients [8,31,32]. Some researchers found that if managers had organizational support, burnout was mitigated [18,33]. Managers also expect staff safety, conflict management, and managing unexpected challenges to be the priority challenges for managers beyond the pandemic [18].

The findings of our study align with other researchers confirming that managers put significant effort into securing workplace safety, ensuring resources, and protecting the physical and psychological health of nurses [11,32]. While our study may not differ, all managers went to great lengths to attend to the psychological and physical safety of staff so they in turn could meet patient care requirements and did so without drawing attention to themselves.

Decision-making is foundational to a managerial role in healthcare and was particularly challenging for managers. Managers found themselves having to pivot early and adapt frequently to the changing work environment to ensure vital and changing information was communicated to frontline staff. The initial waves of the pandemic focused on top-down leadership with little input from managers, and as the pandemic evolved there was more of a shared responsibility in decision-making, especially as it related to patient care at the unit level.

In the initial waves of the pandemic, managers practiced adaptive or flexible leadership to facilitate difficult yet rapid decision-making in their daily activities. Forster et al. (2022) found that health leaders desired flexibility in their work situations as it enabled them to meet different demands and challenges that increased their resilience [34]. Managers reflected on various creative, empathic ways to empower themselves and their staff. For example, managers navigated these situations of uncertainty by thinking outside the box and proposing new ways of doing things rather than returning to some of the “old ways of doing things”. Hartney et al. (2021) found that collective agreements and bureaucratic hierarchical management systems “slow down innovation and change” [8] (p. 1800). In a number of instances, policies were developed and implemented without weaving through a labyrinth of committees or bureaucratic processes, which helped to address the problem more quickly. We argue that the crisis created opportunities for managers to exercise their professional judgement and decision-making for swift problem solving. We recommend that organizational support be reconfigured to make changes more quickly even in non-crisis situations.

Our findings revealed that teamwork and solidarity among managers, their superiors, nurses, and other disciplines facilitated their ability to work together. The uncertainty of working during the pandemic, and how best to navigate the consequences of the pandemic for patient care, improved teamwork. In turn this facilitated trust and support and decreased stress and anxiety, which served as a coping strategy [11]. This aligns with other studies that found working together led to positive outcomes [11,35,36].

Managers made difficult decisions as they struggled with complex intersecting issues including chronic nurse shortages that predated the pandemic. Staff shortages and staff absenteeism aggravated workloads for managers. This was manifested as staff testing positive for the virus or being absent from work due to caring for ill family members. The pandemic compounded these challenges and introduced new concerns such as mandatory overtime, occupational exposure to the virus, and significant moral burden and distress for managers and nurses [37]. The human resource issues exposed gaps in health human resource planning. This leaves nurses remaining in the profession with increased anxiety unless there is clear, decisive, and coordinated action to change the dire situation.

Most of the manager’s time during the pandemic focused on the physical and emotional wellbeing of staff. White (2021) also found managers spent considerable time tending to staff’s emotional wellbeing while also experiencing considerable stress and exhaustion themselves [4]. Organizational support for nurses’ wellbeing can include participatory practices, nurturing communication structures, supporting nurse empowerment, and demonstrating care and concern which have been linked with better nurse performance and competence essential to health-related crises [20,38,39]. However, our findings reveal that, while managers provided physical and emotional care for nurses, and as the pandemic continued through upwards of five waves, nurses’ commitment and turnover intentions increased [13,30].

All managers reported being challenged in managing the unprecedented crisis. Managers revealed they did their best to respond to COVID-19, yet there were minimal reports of managers reporting the need for more training or skills for crisis management. However, managers expressed that better preparation of nurses with the requisite skills and experience are needed for nurse deployment. We recommend managers be involved in refining a disaster preparedness framework to enhance their capacity for complex problem solving and enhanced support for care providers, and possibly contributing to resource inventories to serve as a point of reference in capacity building.

Health organizations call for training programs that build leadership skills to better prepare nurse managers to respond effectively in crisis situations [40,41]. Others affirm this recommendation, stating that there is a need for managers to have the required competencies to better manage staff and patients in an emerging situation [42], as inadequate preparedness may have contributed to increased stress, fear, and difficulty in decision-making [18]. Disaster management preparation for managers and health leaders in hospitals is necessary as the next decade is likely to produce additional global healthcare crises as a result of global warming, international travel, and global connectedness [43]. The need for managers to change leadership practices and engage and empower frontline nurses continues to be recognized as essential to sustaining their emotional health and the health of the workforce, and patients [4,8]. As such, we propose a renewed or extended understanding of organizational resilience in which managers can enhance their ability to lead in the face of inevitable adversities.

### Limitations

The current study is limited in the following ways. First, the study was carried out in the early waves of the pandemic which may have led to different perceptions and variabilities of individual perspectives at the time of the interviews. Second, we interviewed frontline managers but did not take into consideration nurses at the front line of care to hear their experiences and determine how decisions made by managers affected their ability to provide care. In subsequent studies currently being conducted, we are engaging nurses and managers to co-create a path forward to foster a safe, healthy, and supportive work environment that fosters nurses’ psychological health and wellbeing. Finally, all managers were female, which may limit the generalizability of the findings.

## 5. Conclusions

These findings help validate and expand upon an emerging body of literature regarding challenges frontline managers faced during COVID-19. This study found frontline managers faced considerable leadership challenges in the initial waves of the pandemic and took opportunities to support nurses to deliver best possible care and focused on relationships in caring for others that fostered organizational resilience. Managers’ voices need to be heard to better support nurses at the front line of care, given the dire nursing shortage and nurse retention issues. Understanding managers’ experiences can facilitate planning and preparing managers for ongoing and future health crises and strengthening their ability to improve nurses’ working conditions. Training can equip managers with improved leadership and management strategies to be better prepared to respond to future crises. Skillful crisis leadership is a distinct priority needed to manage and sustain nurses’ mental health and wellbeing, while striving to improve organizational resilience during pivotal times.

## Figures and Tables

**Table 1:** Semi-structured interview guide.

**General statement**: We are meeting today to talk about your experience as a manager during COVID-19 and how organizational resilience was activated in your health care setting, so that we can learn from this for ongoing and future responses to health care crises.
**Introductory question**
When you heard the phrase that a “global pandemic was declared” what did this mean to you as a manager?
**Objective 1:** Describe the leadership challenges identified by senior health leader in the context of the COVID-19 crisis.
➢ Tell me how you learned about how the pandemic was going to affect your unit(s)?
➢ How did you begin preparing your staff for the COVID-19 response?
➢ How did you prepare yourself, if at all, as a manager for this crisis?
**Objective 2:** Describe how senior healthcare leaders worked with key stakeholders (hospital, regional, government) to make decisions and deploy resources (human and non-human) when taking organizational action to reduce the impact of COVID on staff and patients.
➢ How did senior leaders engage with you so you could begin responding to the crisis?
➢ What were some of the pressures you faced? How did you respond to these pressures?
**Objective 3:** Determine how senior leaders’ decision-making influenced front-line managers ability to respond to their decisions and shape staff and patient outcomes.
➢ What key decisions were made by senior leadership that impacted your unit the most?
➢ How did you respond to senior leadership’s decisions that affected the operation of your unit(s)?
➢ How did senior leadership’s decisions affect staff and their ability to care for patients?
➢ How did senior leadership support you?
➢ What strategies did you use to overcome difficult situations on the unit? How did others react?
**Objective 4:** Identify the lessons learned that can enhance organizational resilience in anticipation of managing future healthcare crises.
➢ How was your unit different or the same following the crisis? If different, how?
➢ Was the organization able to “bounce back” and improve following the pandemic? If so, explain what this means to you.
➢ Post COVID-19, how, if at all, did new behaviors and practices of leaders and managers change the way the unit and hospital operates?
➢ What did you learn as a manager from this crisis? What would you do differently in future crises?
**Final question:** Is there anything else you’d like to share with me regarding your experience as a manager during COVID-19 or how your unit or organization bounced back from the crisis?

**Table 2:** Demographics of participants.

	Number	Percentage
**Age**	**(n = 14)**	
21–30 years	0	0.00
31–40 years	3	21.43
41–50 years	6	42.86
51–60 years	5	35.71
**Gender**	**(n = 14)**	
Female	14	100.00
Male	0	0.00
**Education**	**(n = 14)**	
Only Nursing Degree	4	28.57
Social Work Degree	3	21.43
Degree in Nursing with Certificate	2	14.29
Degree and Diploma in Nursing	2	14.29
Diploma in Nursing with Certificate	1	7.14
Diploma Only	1	7.14
Unspecified Degree Only	1	7.14
**Years of Experience in** **Frontline Manager Role**	**(n = 14)**	
<5 years	6	42.86
5–9 years	1	7.14
10–14 years	3	21.43
15–19 years	2	14.29
20–35 years	2	14.29
**# of Individuals Supervised**	**(n = 14)**	
<50	5	35.71
51–100	7	50.00
100–150	2	14.29
**Department/Unit**	**(n = 14)** *	
Dialysis	1	7.14
Ambulatory Care Clinic	1	7.14
Emergency Care Clinic	1	7.14
Intensive Care	1	7.14
Long Term Care	1	7.14
Observation Unit	1	7.14
Geriatric Rehab	1	7.14
Public Health	1	7.14

* Counts and percentages may not add up to n = 14 or 100% due to some participants indicating more than one answer and some participants choosing not to answer.

**Table 3:** Narratives linked with the sub-themes within each theme.

Leadership Challenges	Participant Narratives
Inadequate physical resources	Even with N95 masks … I had to advocate to make sure that we had the proper N95 masks on XXX unit because we haven’t had a lot of cases, but we still need to have the supply of masks in case we do get a patient. And so, you know, if you get a patient on a weekend and you’ve got no supplies and everyone else’s supplies are on lockdown and they’re not going to share, you put staff at risk, so we still need to have a plan in place. (HCP07)
Emotional distress	Of course, as the pandemic has gone on, there’s also the factor of adding in fatigue and burnout and the frustrations with things going on longer and longer and longer. So, I feel the team support is still there, but you can tell that so is exhaustion, and so is frustration and spiritual distress. All those kind of things are showing up in the staff and still trying to support them now in different ways. (HLCRP08)
Influence of senior leader decision making on managers	
Constant change became a normal part of the workplace	It was a precarious situation because one week we were saying, you know, “Don’t wear masks to work, you don’t have to wear masks” and we were talking about how to manage the people who were insisting on wearing masks and how that might be an HR issue and it was very adamant that, “No, you do not wear masks into work” and then the next week it was like, “No, you must wear a mask.” It was so crazy-making, right? It was like, you know, on Monday, “This paper is white, you do not say that it’s black. It is a white piece of paper” but then by Friday, “No, this a black piece of paper, you do not say it’s white”. So was very challenging to keep your credibility as a leader and as a manager as the information and the rules changed really quickly. You looked like a buffoon and you looked like you were just following the rules and not questioning the logic. (HLCRP22)
Shortage of human resources	We all wish, it would’ve been nice to have some extra positions and movement, but I can’t say they [senior leadership] didn’t support it, but I just feel a lot of people were and are still doing a lot of extra jobs to try and cover all the extra needs. And they talked about redeployment and it’s not like they weren’t talking about it, but with [anonymous facility], I mean long-term care was obviously having some staffing issues and then making sure seniors were all hydrated, so then we redeploy our health care aides but then that puts an excess strain on the nurses. Like I would say they [senior leadership] were supportive, but they weren’t able to provide extra bodies. (HLCRP13)
Adapting the delivery of care	I think that priority issues was kind of twofold. I mean, we were getting provincial direction and—from a governing table around what the strategic priorities were for the organization, from a provincial perspective. So it was coming that way from a top down delivery. But then within the organization or the facility, it was prioritizing the needs as they came on a day to day basis, so sort of that operational decision making around what was a priority that day sometimes or that moment. And we were dealing with things like outbreaks that were unexpected, and so that would become the priority of the day. So, there was many circumstances where something that came up superseded a plan priority. (HLCRP29)
Cooperation and collaboration	We were really fortunate, and I think we’re a little bit unique in the [region E]. We’re geographically gigantic but population wise we’re only about 77,000 people that live in our region. I know our CEO. Like I can call her anytime I want. She actually set up meetings that she had weekly with all of our management teams where we all met. So, it was all different parts of our team. It was like finance, meeting with infection control, meeting with our physicians, everybody meeting together to discuss what’s been happening and how are we going to manage this. (HLCRP09)
Lessons learned	
Managerial caring behaviours and role modelling	And for me, it was really important that anything that I asked my team to do I was willing to do. So if my team was going out to extra personal care home outbreaks, I was going to extra personal care home outbreaks. So I had been that person sanitizing stair railings because that’s what I needed to do that day. So yeah, making sure that I was willing to do the work that they were also willing to do. (HLCRP26)
Adaptive leadership	We thought outside the box and came up with better ways of utilizing the equipment we did have. And some of the equipment that was sent we were able to use in some ways here. But some of it we just didn’t use. I think, essentially, we did whatever we could do within the limitations of our facility and made the best—did the best we could with the resources we were given. (HLCRP10)
Training and education	I’ve learned that change management is way more important than I ever thought it was. Again, we have always talked change management, right? So we’ve always talked about how we need to be ready as organizations for strat-plan changes and how we need to be ready as organizations for amalgamations, and how we need to be ready as organizations to do that type of work, and so have a little bit of change management abilities. But I would say that that is one thing that glared through is that the changes were so rapid that there was no time to catch your breath in between them. There was actually no time to feel confident and comfortable in the change before you moved on to the next thing…. change management is very important, but it’s definitely not linear. That we definitely bounce around a lot. Definitely look to people’s stages of change with them as to where they were at in all of this. Yeah, I think it’s time to shake up how our managers are trained in terms of change management, because we did not have training to meet that need. (HLCRP26)
Culture of care for self and staff	A lot of people who had been picking up [shifts] trying to help with sick time, I would take them inside and I would thank them for doing that. But also I’d be like, you do need to have personal time. And I would say, I appreciate you picking it [shift] up but please don’t pick up so much that you’re going to make yourself sick and not able to come to work. I did have quite a few discussions with a lot of people—because we were asked to remind staff, if you work—pick up when you can. And I’d always say to these people if you can’t pick up I understand why you can’t pick up. We’re at a point where your mental capacity to come to work is—you’re full. And you need to go home and recharge. During COVID we all know there was a lot of mandating, right? A lot of shifts unfilled. And I did my best to not mandate some of the strategies—I would make deals with people. If you work this shift I’ll give you off this one, right? Try to balance out the days with needs, so staff wouldn’t feel scared to come to work wondering, am I going to be staying for 16 h today? When do I get to go home? Because that was a real fear at some point. And it’s actually still to this day it’s still a fear right? (HLCRP28)

## Data Availability

The data presented in this study are available on request from the corresponding author. The data are not publicly available due to ethical and privacy restrictions.
